# Supplementary material for: Decreased Expression of Aquaporins as a Feature of Tubular Damage in Lupus Nephritis
Source: Cells. 2025 Mar 5;14(5):380. doi: 10.3390/cells14050380 (PMC11899336; doi:10.3390/cells14050380)
Supplement: Supplementary file 1 [file cells-14-00380-s001.zip › cells-3444520-supplementary.pdf]

Supplementary data

Table S1. Clinical subclassification and comorbidities.

eGFR, estimated glomerular filtration rate; m, month; g, grams; LN, lupus nephritis.

**Proteinuria**	
Nephrotic range at diagnosis (NR)	8/37
25% decrease at 3 m	19/27 (1 NR)
50% decrease at 6m	11/26 (1 NR)
< 0,5 g/g at 1-year	15/26 (2 NR)
**eGFR at 1-year**	
>90	18/30
90-60	5/30
15-60	2/30
<15	4/30
**eGFR at diagnosis**	
>90	18/35
90-60	6/35
15-60	9/35
<15	2/35
**Comorbidities**	
Previous LN (> 2 y)	4/37
Diabetes	1/37
Hypertension	5/37
Tobacco	2/37

Table S2. Clinical data from healthy controls

Age and sex were missing for one sample, comorbidities were not available for sample obtained prior to engraftment.

**Clinical data**	
Age (years)	55 [43-57.5]
Female/Male ratio	7/1
**Comorbidities**	
Tobacco	0/6
Diabetes	0/6
Hypertension	5/6
**Reason for biopsy**	
Prior to engraftment	3/9
Autopsy	2/9
Nephrectomy for localised renal carcinoma	4/9

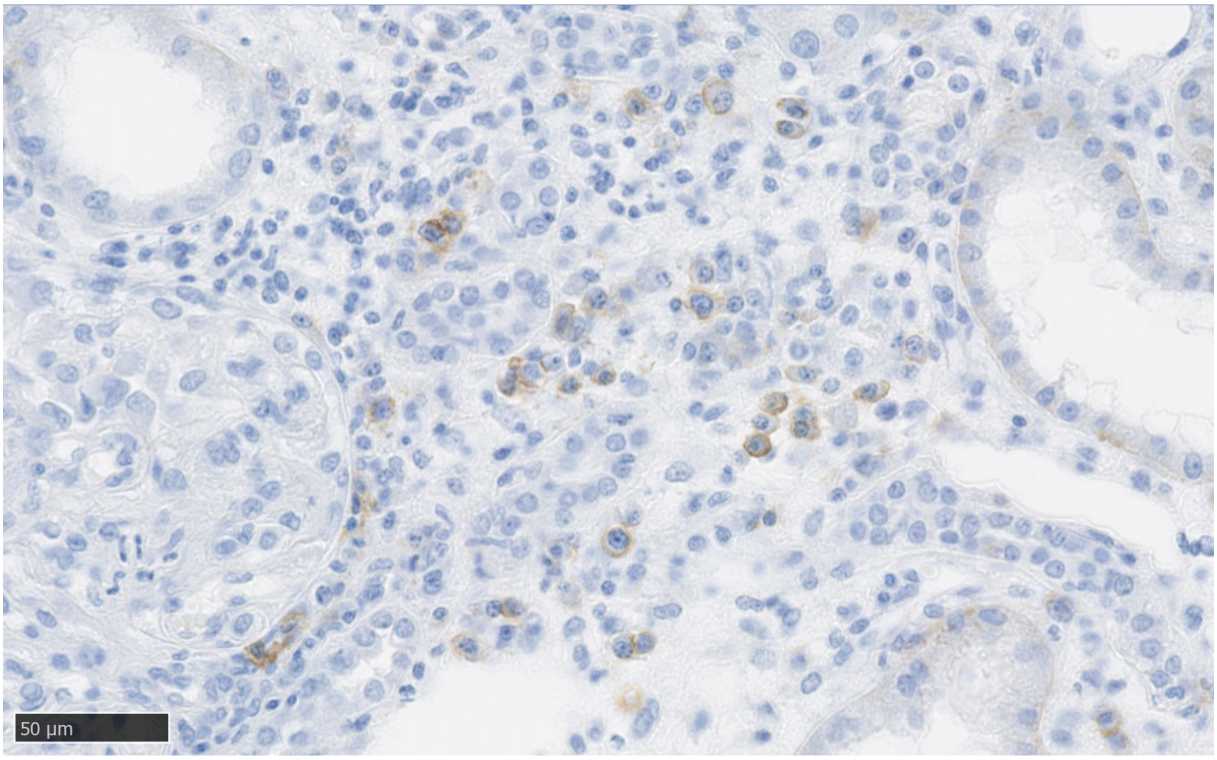

Figure S1. Interstitial monocellular infiltrate expressing aquaporin 3, field magnification 40x.

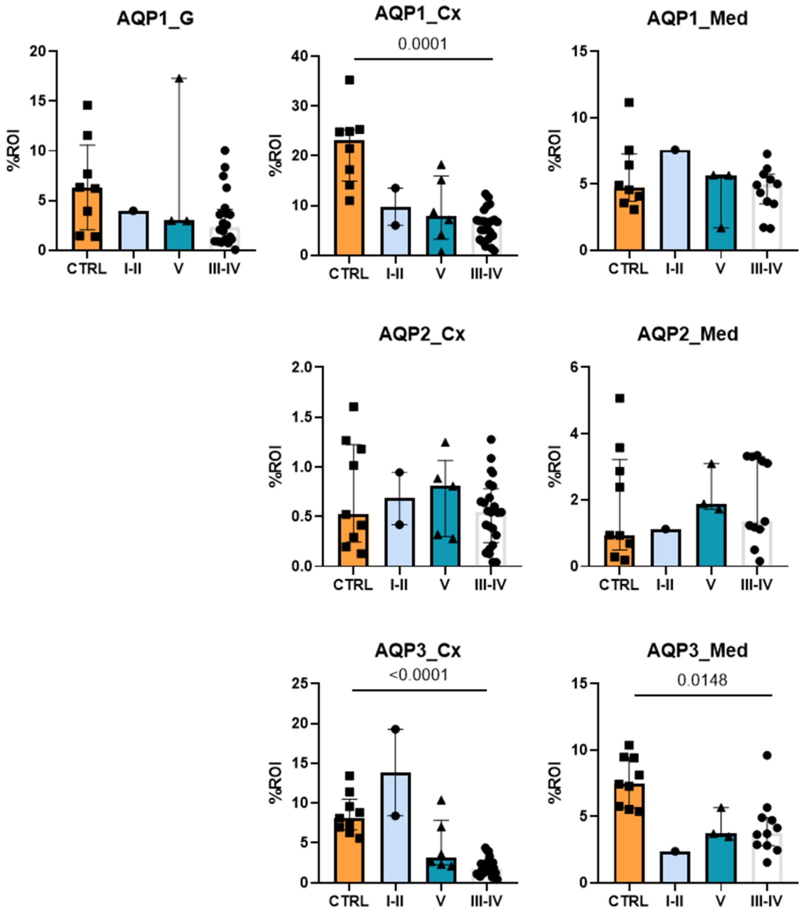

Figure S2. Proportion of staining in the region of interest (%ROI) in healthy controls and LN subgroup according to ISN/RPS 2018 classification.

Results are expressed as the median with interquartile range of %ROI, tested by Kruskal-Wallis with Dunn's multiple comparison test; corrected p-values are depicted on graphs.

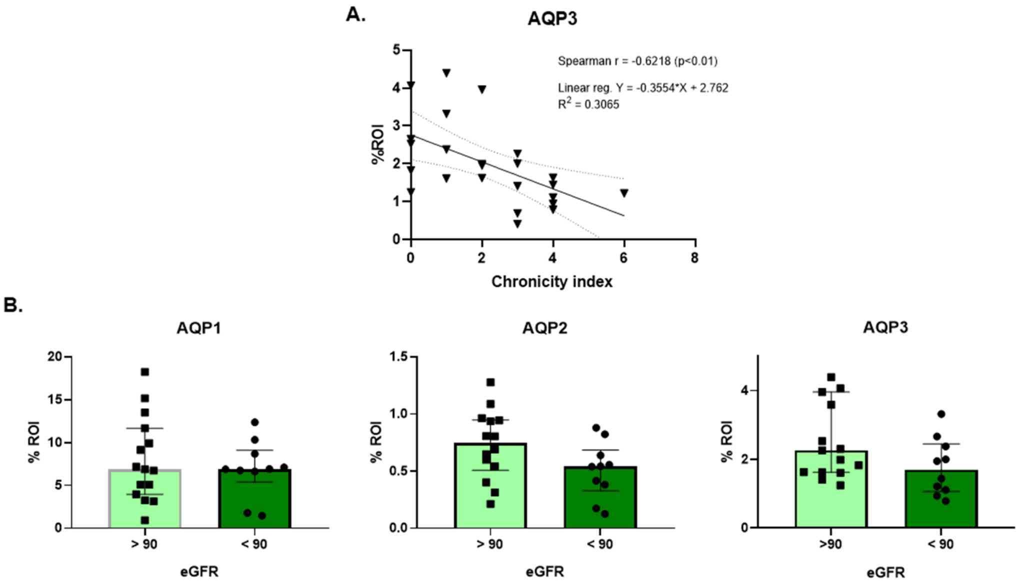

Figure S3. A. Correlation between NIH chronicity index and cortical expression of AQP3 with linear regression test **B.** Relationship between aquaporin expression in the renal cortex and presence of renal insufficiency 1 year after biopsy evaluated by estimated glomerular filtration rate (eGFR) under 90 milliliter by minutes per 1,73 m²

Results are expressed as the median with interquartile range of %ROI.

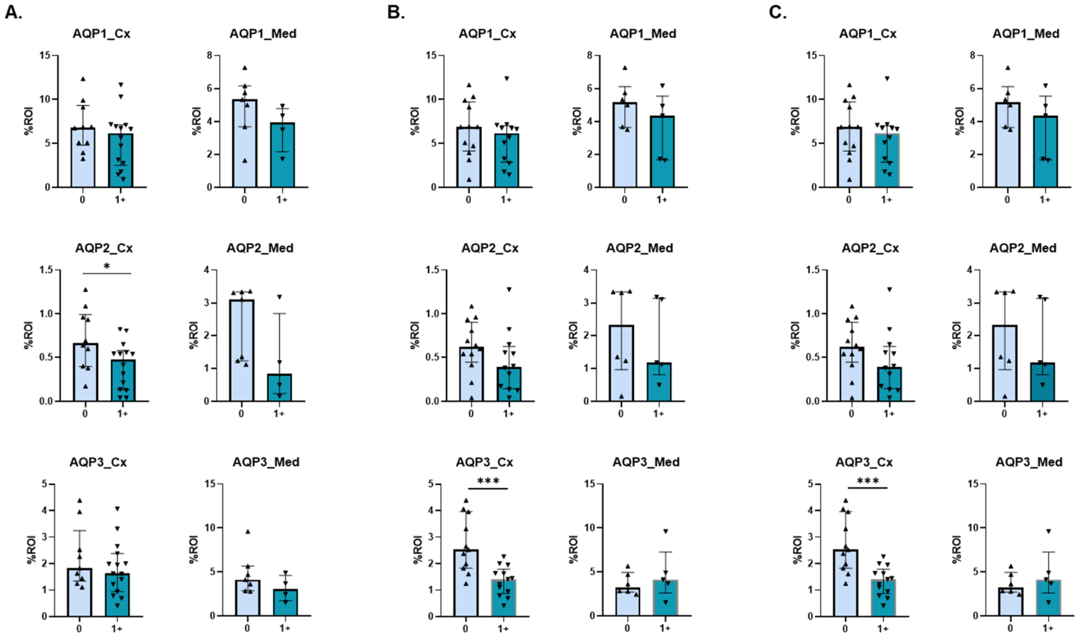

Figure S4. Proportion of staining in cortex (Cx) or Medulla (Med) (%ROI) according to absence (0) or presence (score of 1 or more) of (A.) interstitial inflammation according to Interstitial leukocyte score (B.) tubular atrophy or (C.) interstitial fibrosis.

Results are expressed as the median with interquartile range of %ROI tested by Mann-Whitney; *: $p<0.05$; ***: $p<0.001$.
